# Population Genetics of a Trochid Gastropod Broadens Picture of Caribbean Sea Connectivity

**DOI:** 10.1371/journal.pone.0012675

**Published:** 2010-09-10

**Authors:** Edgardo Díaz-Ferguson, Robert Haney, John Wares, Brian Silliman

**Affiliations:** 1 Department of Biology, University of Florida, Gainesville, Florida, United States of America; 2 Department of Organismal Biology and Anatomy, University of Chicago, Illinois, Chicago, United States of America; 3 Department of Genetics, University of Georgia, Athens, Georgia, United States of America; Northeastern University, United States of America

## Abstract

**Background:**

Regional genetic connectivity models are critical for successful conservation and management of marine species. Even though rocky shore invertebrates have been used as model systems to understand genetic structure in some marine environments, our understanding of connectivity in Caribbean communities is based overwhelmingly on studies of tropical fishes and corals. In this study, we investigate population connectivity and diversity of *Cittarium pica*, an abundant rocky shore trochid gastropod that is commercially harvested across its natural range, from the Bahamas to Venezuela.

**Methodology/Principal Findings:**

We tested for genetic structure using DNA sequence variation at the mitochondrial COI and 16S loci, AMOVA and distance-based methods. We found substantial differentiation among Caribbean sites. Yet, genetic differentiation was associated only with larger geographic scales within the Caribbean, and the pattern of differentiation only partially matched previous assessments of Caribbean connectivity, including those based on larval dispersal from hydrodynamic models. For instance, the Bahamas, considered an independent region by previous hydrodynamic studies, showed strong association with Eastern Caribbean sites in our study. Further, Bonaire (located in the east and close to the meridional division of the Caribbean basin) seems to be isolated from other Eastern sites.

**Conclusions/Significance:**

The significant genetic structure and observed in *C. pica* has some commonalities in pattern with more commonly sampled taxa, but presents features, such as the differentiation of Bonaire, that appear unique. Further, the level of differentiation, together with regional patterns of diversity, has important implications for the application of conservation and management strategies in this commercially harvested species.

## Introduction

Marine reserves are a primary mechanism to deal with threats facing populations and species in marine biodiversity hotspots such as the Caribbean [1], [2], and the effective design of marine reserves relies heavily on information about the connectivity of populations [3]. Genetic data is especially important in marine systems as it allows inferences to be made regarding connectivity that are often difficult to obtain by other means [4]. Data on genetic structure and variability is key for the establishment of population boundaries and for monitoring populations under management [3], [5], [6], and integration of this data into management and conservation plans has been deemed essential for the appropriate establishment of connective corridors, conservation units and common nation protected areas [3], [6]–[8].

In the Caribbean marine province, four connectivity regions have been proposed based on complex hydrodynamic current models: Eastern Caribbean; Western Caribbean; Bahamas and Panama–Colombia [9]. Dispersal is extensive within these regions, but dispersal across their boundaries is lacking. Some aspects of this regional arrangement are supported by genetic data from marine taxa. For example, phylogeographic breaks in the goby *Elacatinus evelynae*, which support isolation of the Eastern and Western Caribbean regions across the Mona passage (between Puerto Rico and Hispaniola), as well as the differentiation of populations in the Bahamas from those to the south across the Exuma Passage [10]. In Elkhorn Coral *Acropora palmata*, Eastern and Western Caribbean populations appear to be genetically differentiated [11]. Isolation of populations in the Eastern and Western Caribbean across the Florida peninsula has also been documented [12], [13].

However, efforts to define connectivity through population genetic structure have been focused overwhelmingly on coral and fish populations [10], [14], [15]. In other well-represented marine ecosystems in the Caribbean basin, data on genetic structure and diversity are lacking. For example, although tropical rocky shore ecosystems have traditionally been considered models for the study of recruitment patterns, vertical zonation, community structure and trophic interactions [16]–[19], there is comparatively little work on genetic variation. In fact, on Caribbean rocky shores genetic information is limited to the sea urchins *Echinometra lucunter* and *Eucidaris* [20], [21]. In contrast, there are numerous genetic studies of rocky shore ecosystems in temperate zones [22]–[26]. To begin examining the Caribbean rocky intertidal in this regard, we use as a focal species the West Indian topshell (*Cittarium pica*), a trochid gastropod that is a key primary consumer, distributed throughout the West Indies and on Central American shores where it inhabits crevices and small holes of exposed areas [27]–[29].

The population structure of this species is of special interest due to a short pelagic larval duration (PLD; 3.5–4.5 days) which greatly limits the potential for dispersal, and because of economic significance, as it is commercially harvested [27], [30]. In fact, *C. pica* has been included in the red list of endangered species of several Caribbean countries and territories, including Colombia, the U.S. Virgin Islands, Bermuda and Dominica [28], [31], and is protected in Bermuda and the Virgin Islands [32]. Furthermore, a conservation and management plan for the species is currently being executed in the U.S. Virgin Islands and Puerto Rico [33]. However, in Southwestern regions where *C. pica* is harvested for local consumption and sold as handcraft for tourists a ban for this species has not yet been established [33]. Given the threat to this species from commercial exploitation, this research will make available valuable genetic data that can be used in the establishment of regional conservation policies and networks, which is fundamental for the sustainability of the topshell fishery.

Here we attempt to answer several questions related to the phylogeography and population genetics of *C. pica* through the analysis of mitochondrial DNA sequence variation. First, what degree of genetic structure occurs in *C. pica* and at what spatial scales? Do differentiated populations coincide with expectations from hydrodynamic dispersal models? Are phylogeographic breaks detected in *C. pica* consistent with previous data from other taxa and ecosystems, which would suggest a common underlying basis for differentiation? The existence of common breaks for Caribbean taxa supports the role of vicariance in the origins and evolution of Caribbean marine communities. Although the correlation between pelagic larval duration (PLD) and genetic structure is not always tight [8], species with very short PLD, such as *C. pica*, often exhibit strong genetic structure. In marine animals, the extent of realized dispersal and gene flow are strongly influenced by mesoscale oceanography, the duration of planktonic larval development and larval behavior [34]–[36]. However, the importance of these factors varies among taxa and among geographic regions [8], [9], [37]. We examine the evidence for their contribution in mediating connectivity among Caribbean populations of *C. pica*. Lastly, does a spatial trend in diversity levels occur in *C. pica*? For fishes and corals, because western Caribbean locations have more extensive coral reef ecosystems that may support larger population sizes, these regions are expected to have elevated genetic diversity [2], [36]. Spatial patterns of genetic diversity can also provide insight into the biogeographic origins and large-scale historical demography of this species. The acquisition of this information is not only essential for conservation and management, but also for understanding population dynamics and community assembly in Caribbean rocky shore ecosystems [35], [38], [39].

## Materials and Methods

### Study Site and Sampling

**Figure 1:**
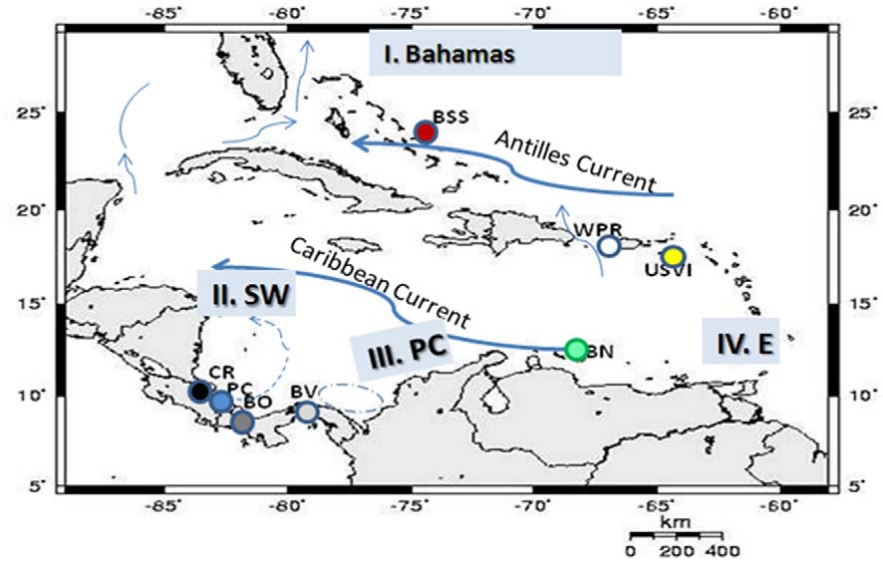
Sampling locations and regions for *C. pica* in the Caribbean. Region I- **Bahamas** [**BSS:** San Salvador Bahamas], Region II- Southwestern **SW** [**CR:** Punta Cocles, **PC:** Playa Chiquita, **BO:** Bocas del Toro], Region III-Panama central **PC** [**BV:** Buenaventura-Colon-Panama], Region IV- Eastern **E** [**BN:** Bonaire, **USVI:**Virgin Islands, **WPR**: Puerto Rico (Guajataca, Aguadilla and La Parguera)].

The Caribbean region spans from an upwelling zone near Cape Canaveral, Florida in the north to the fresh water outflows of the Amazon and Orinoco rivers in the south, and from Central America in the west to the Atlantic Ocean in the east [40]. Within this geographic area, rocky shores are common and widely distributed. Our sampling scheme was designed to test whether the boundaries of hydrodynamic connectivity regions previously defined [9] were broadly coincident with genetic breaks in *C. pica*. We sampled small crevices in well-exposed rocky intertidal areas where *C. pica* is commonly found within each region defined by [9]. Sites sampled in each region include: **region I. Panama-Colombia [**Central Panama Buenaventura (BV), Island at Colon province 9.315°N, 79.401°W]; **region II. Southwestern Caribbean [**Western Panama, Isla Bastimentos (BO) 9.175°N, 82.080°W, Southern Costa Rica, Punta Cocles (CR) 10.458°N, 83.203°W and Playa Chiquita (PC) 9.382°N, 82.424°W]**; region III. Eastern Caribbean [**Bonaire-Netherland Antilles (BN) 12.120°N, 68.162°W, US Virgin Islands, Saint Croix (USVI) 17.412°N, 64.413W, La Parguera-Puerto Rico 17.555°N, 67.113°W, Aguadilla-Puerto Rico (WPR) 18.255°N, 67.092°W]; **region IV. Bahamas** [San Salvador, Bahamas (BSS) 24.061°N, 74.291°N] (Table S1, Figure 1). A total of 157 adult individuals of *C. pica* were collected among these regions, with up to 25 adult *C. pica* specimens sampled per site (average sample: n = 15 per site). Individuals were collected during open fishing season for the species and hence permits were not required for the majority of sample locations. In Bonaire National Marine Park (STINAPA), samples were collected by local biologists with an authorization letter (intern permit) from the director of the park.

### Data Collection

DNA was isolated from sampled individuals by using either a QIAGEN DNeasy kit (Qiagen, Rockville, MD), or a cetyl trimethylammonium bromide (CTAB) isolation protocol [41]. PCR amplifications of two mitochondrial fragments, 16S rRNA (primers AR and BRS: annealing temperature 50°C) (Palumbi 1996) and COI (primers HCOI and LCOI: annealing temperature of 45°C) [42], [43] were conducted. PCR products were cleaned with a QIAGEN PCR purification kit and sequenced on an ABI-PRISM sequencer. Sequence products were edited and aligned using Codon Code Aligner 2.0.4. and Geneious Pro 4.7.5.

### Population Structure and Genetic Connectivity

A median joining algorithm was used to build a minimum spanning network to examine the evolutionary relationships among haplotypes, as implemented in Network 4.5 (http://fluxus-engineering.com/). This method combines features of Kruskall's distance-based algorithm for finding minimum spanning trees with maximum-parsimony [44].

Population differentiation among sites were examined using pairwise F_ST_ values based on a Kimura 2-parameter distance calculated in Arlequin 3.11 [45], with 1000 permutations of haplotypes among populations used to construct a null distribution for significance testing. An analysis of molecular variance (AMOVA) was implemented in ARLEQUIN 3.11 to test whether a significant proportion of genetic variation at both loci in this study was partitioned among the connectivity regions of [9].

We also used SAMOVA 1.0 [46] to define the group structure that maximized the among groups variance component for both loci via a simulated annealing procedure, which was subsequently tested for significance *a posteriori* in ARLEQUIN 3.11.

### Genetic Diversity and Demographic History

Summary statistics, including the number of alleles, nucleotide diversity (π) and the neutrality tests Tajima's D [47], hereafter referred to as D_T_, and Fu's F_s_ [48] were calculated for each locus at each site using DNAsp. v.5.0 [49]. Under neutrality and demographic equilibrium D_T_ will be approximately zero, and will be negative under certain demographic and selective scenarios, including population expansion, as will Fu's F_s_. Fu's F_s_ is a particularly useful test statistic as it appears to be the most sensitive to population expansions [50]. Significant deviations from neutral expectations for both statistics were confirmed by using standard coalescent simulation methods, also implemented in DNAsp v.5.0 (1000 replicates).

## Results

### Basic Features of the Molecular Data

After exclusion of sequences with 20% of their total length or more constituting missing or low quality data with PHRED score <20 [51], a total of 106 COI sequences (384 bp) and 126 sequences of 16S rRNA (351 bp) were analyzed from *C. pica*. A total of 37 distinct haplotypes were present among the 106 COI sequences, and 38 distinct haplotypes were found in the 16S sequence data. Overall values of nucleotide and haplotype diversity were substantial for both COI (h = 0.937 ; π = 0.015) and 16S (h = 0.911; π = 0.017).

### Phylogeographic Structure and Genetic Connectivity

**Figure 2:**
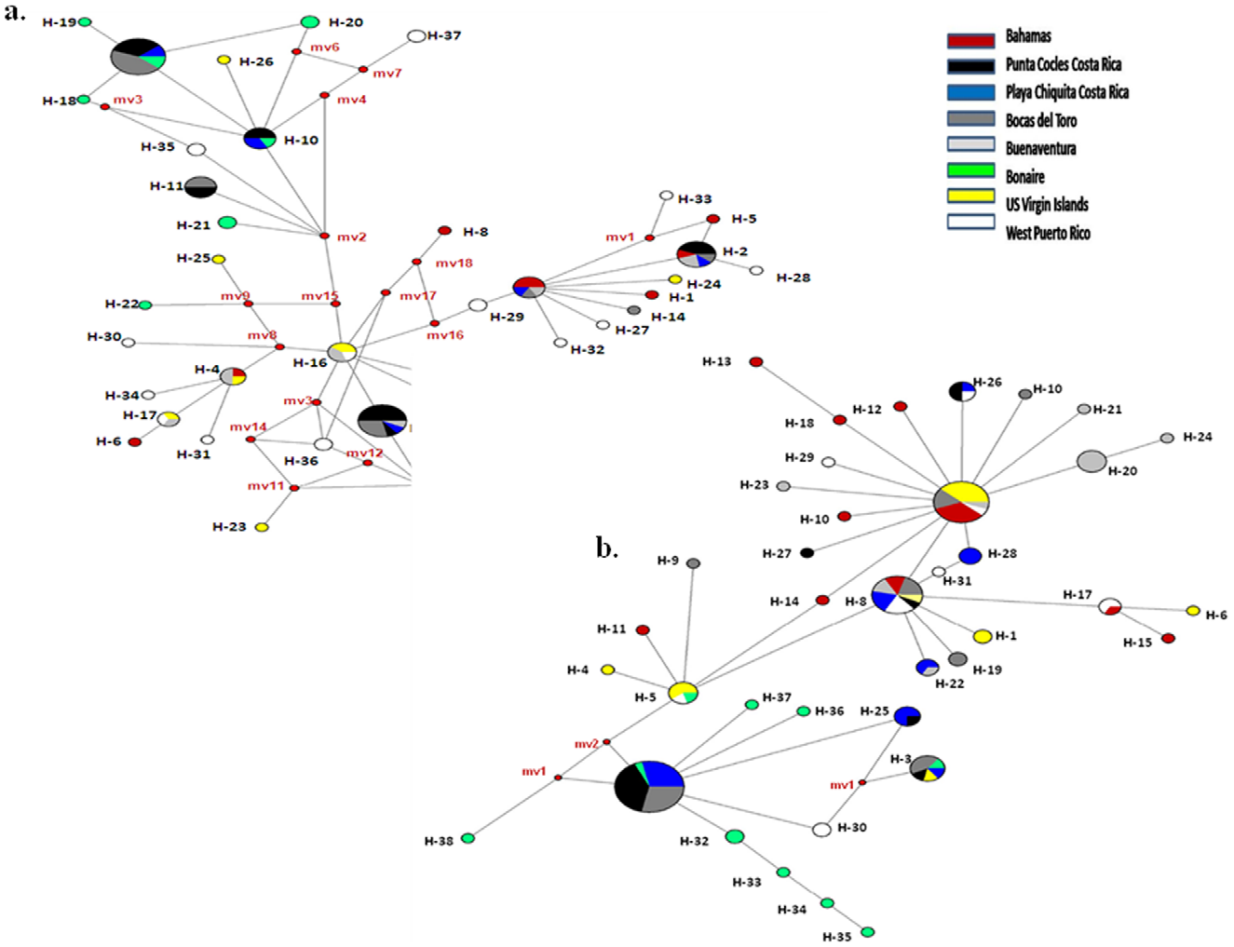
Median-joining networks for COI (a) and 16S (b) *C. pica* haplotypes. Each haplotype is labeled and represented by a circle and its area is proportional to its relative frequency by location. For a given haplotype, color coding indicates in what proportion it is found at different sites. Numbers correspond to the positions of mutations occurring in the studied COI and 16S fragments. Median vectors (**mv**) are represented as small red circles. The sequences (haplotypes) by site have GenBank Accession Nos *GU726381*-*GU726460* (COI) and *GU733509-GU733607* (16S).

Haplotype relationships were visualized by median joining networks (Figure 2). Although haplotypes from individual sites or geographic regions do not form exclusive clades, and sharing of haplotypes across sites and regions does occur to some extent, clear patterns of endemism are seen in both genes. In particular, 70.3% of COI haplotypes and 76.3% of 16S haplotypes are restricted to single sites.

Genetic differentiation of sites and regions is corroborated by F_st_ values. The overall F_st_ is 0.168 for COI, corresponding to an effective number of migrants per generation (N_f_m) of 2.48 under an island model of migration [52]. Overall F_st_ was 0.358 for 16S, corresponding to an N_f_m of 0.900. Exactly 50% of pairwise comparisons among sites for COI are significant (p<0.05), while 82% are significant for 16S, likely due in part to generally larger sample sizes for 16S (Table 1). Results are similar if the analysis is performed using 76 individuals for which both COI and 16S were sequenced (not shown). In spite of the strong genetic structure evident, both loci are consistent in indicating a lack of differentiation among nearby sites (<300 km of separation) occurring within the regional boundaries proposed by Cowen et al. (2006), such as USVI and WPR and the three Southwestern sites (CR, PC, BO). Of comparisons among sites in different regions, 66% were significant (50% of comparisons in COI, 82% in 16S). This was true even in comparisons of nearby sites. For example, strong differentiation occurs between the Central Panama (BV) and Southwestern sites despite their relative proximity (<470 km).

**Table 1:** Triangular matrices of pairwise F_st_ values for COI and 16S between populations.

*SITE*	BSS	CR	PC	BO	BV	BN	USVI	WPR
BSS	0	0.2523**	0.2090*	0.2896**	0.0003	0.5236**	0.0680	0.0368
CR	0.6186**	0	−0.0734	−0.0349	0.1947**	0.1742*	0.1058	0.0849*
PC	0.3624****	0.0533	0	−0.0767	0.2113*	0.1150	0.0931	0.04459
BO	0.3751**	0.0466	−0.0400	0	0.2463**	0.1154	0.1370*	0.1129*
BV	0.0997*	0.6469**	0.3965**	0.4082**	0	0.5325**	−0.0140	0.0029
BN	0.6948**	0.0701	0.2040*	0.1942*	0.7157**	0	0.3748**	0.3198**
USVI	0.01823	0.5242**	0.2513**	0.2667**	0.1354**	0.6152**	0	−0.0136
WPR	0.1060*	0.3553**	0.0946	0.1100	0.1827**	0.4654**	−0.0021	0

**Note:** All p-values were determined with 1000 permutations of haplotypes among populations and significance is indicated with one or two asterisks (*p<0.05 and **p<0.01). Values for COI are in the upper triangular matrix and those for 16S are in the lower matrix. Refer to Figure 1 for locality abbreviations.

**Figure 3:**
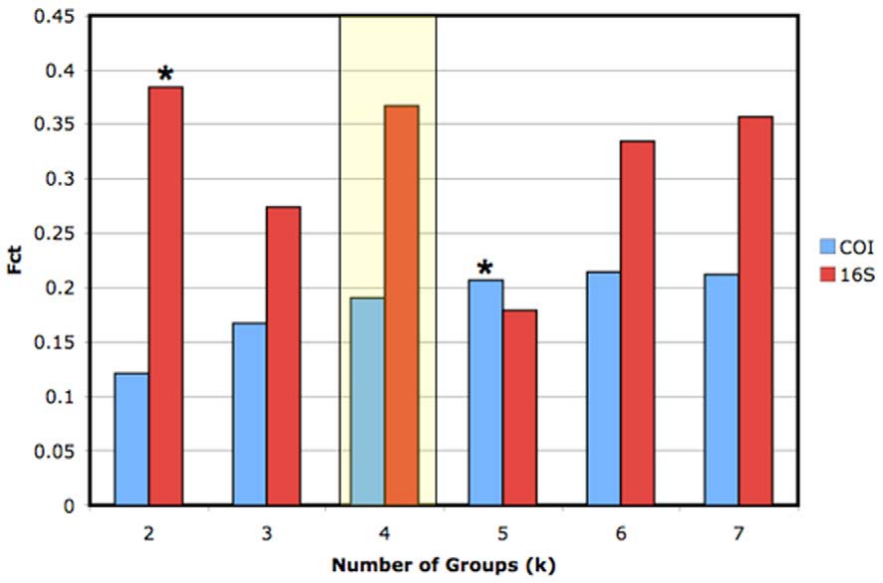
Values of F_CT_ for alternative numbers of groups in SAMOVA for COI and 16S. * indicates the maximal F_CT_ groupings based on the COI and 16S data, while the shaded region delineates the structure expected from hydrodynamic models of larval dispersal in the Caribbean.

Yet, an AMOVA with groups defined *a priori* by the four connectivity regions of Cowen et al. (2006) shows a small and insignificant proportion of the variance at both loci partitioned among these regions (Table 2). To explore alternative groupings of populations that maximize the among group variance component, we calculated F_CT_ for all possible numbers of distinct groups from k = 2 to k = 8 in SAMOVA 1.0. Results indicate maximal variance among groups at k = 6 for COI (F_CT_ = 0.214; p = 0.017) and k = 2 (F_CT_ = 0.384; p = 0.022) for 16S (Figure 3). For COI, one group included three nearby sites in the southwestern Caribbean (CR, PC and BO), with other sites constituting their own groups. Although this pattern maximizes among group variance, some sites placed into their own separate groups are not significantly differentiated at COI (Table 1). For 16S, the maximal F_CT_ grouping was as follows: one group contained the three easterly sites (USVI, BSS, WPR) together with the Central Panama site (BV), while the second group contained the three southwestern sites (CR, PC, BO) together with Bonaire (BN). In contrast to COI, several of the sites grouped together in this analysis are in fact significantly differentiated based on F_ST_. As in both analyses several group number models were similar in the amount of variance explained, we also present the group configurations for these alternative outcomes (Table 3). Significant variance components also occurred between populations within groups and within individual site populations (Table 3).

**Table 2:** AMOVA results for *C. pica* COI and 16S for the four Caribbean connectivity regions [9].

Gene	Source of variation	d.f	% of variation	Fixation index	*p*
**COI**	Among Connectivity Regions	3	6.15	0.0615	0.20332
	Among Populations Within Regions	4	9.75	0.1038	0.00098
	Within Populations	98	84.11	0.1589	<0.00001
**16S**	Among Connectivity Regions	3	12.09	0.12087	0.15054
	Among Populations Within Regions	4	21.82	0.24816	<0.00001
	Within Populations	118	66.10	0.33904	<0.00001

**Note:** Columns indicate sources of genetic variation, the amount of variation accounted for by each source expressed as a percentage, and the fixation indices with associated significance levels determined through 1000 permutations of the data.

**Table 3:** SAMOVA results for COI and 16S.

COI	Source of variation	d.f	% of variation	*p*
*Groups:*1-BN; 2-BSS; 3-USVI; 4-WPR; 5-CR, PC, BO; 6-BVF_CT_ = 0.214	Among Groups	5	21.44	0.017
	Among Populations Within Groups	2	−4.01	<0.00001
	Within Populations	98	82.57	<0.00001
*Groups:*1-USVI; 2-BSS; 3-BN; 4-CR; 5-PC, BO; 6-BV; 7-WPRF_CT_ = 0.212	Among Groups	6	21.21	0.031
	Among Populations Within Groups	1	−6.07	<0.00001
	Within Populations	98	84.86	<0.00001
*Groups:*1-BSS; 2-USVI, WPR; 3-BV; 4-CR, PC, BO; 5-BNF_CT_ = 0.206	Among Groups	4	20.66	0.005
	Among Populations Within Groups	3	−2.77	<0.00001
	Within Populations	98	82.11	<0.00001

16S	Source of variation	d.f	% of variation	*p*
*Groups:*1-USVI, BSS, BV, WPR; 2-BO, CR, PC, BNF_CT_ = 0.384	Among Groups	1	38.4	0.022
	Among Populations Within Groups	6	4.79	<0.00001
	Within Populations	118	56.81	<0.00001
*Groups:*1-BO, CR, PC; 2-BN; 3-BV; 4-USVI, BSS, WPRF_CT_ = 0.367	Among Groups	3	36.7	0.002
	Among Populations Within Groups	4	1.61	<0.00001
	Within Populations	118	61.69	<0.00001
*Groups:*1-BV; 2-USVI; 3-BSS; 4-BO, PC; 5-CR; 6-WPR; 7-BNF_CT_ = 0.357	Among Groups	6	35.68	0.041
	Among Populations Within Groups	1	−2.27	<0.00001
	Within Populations	118	66.58	<0.00001

**Note:** Groups of sites were defined based on maximization of between groups variation. As three alternative configurations occurred for both loci with similar values of F_ct_, all three are presented in order of descending F_ct_. Columns indicate sources of genetic variation, the amount of variation accounted for by each source expressed as a percentage, and the fixation indices with associated significance levels determined through 1000 permutations of the data.

### Genetic Diversity and Demographic History

For COI among sites, π values ranged between 0.009 and 0.017 (π_avg_  = 0.013), and *h* values ranged between 0.784 and 0.972 (*h*_avg_  = 0.909). The highest values of π for COI were identified in Eastern sites (WPR and USVI) and the Bahamas (BSS), whereas the lowest values were observed in BN and BV with Southwestern sites (CR, PC and BO) intermediate (Table S1). The highest *h* values were also recorded in Eastern sites and BSS, while Southwestern sites showed substantially lower values of *h* at COI, with BV and BN intermediate.

For 16S, among sites π ranged between 0.004 and 0.017 (π_avg_ = 0.012), while *h* ranged between 0.581 and 0.982 (*h*_avg_  = 0.836). However, in contrast to COI the highest values of π for 16S were found in Bonaire (BN) and in Southwestern sites (PC, BO) with the lowest values in BV and BSS (Table S1). Similar to COI the highest values of *h* for 16S occurred in Eastern sites, along with Bonaire and the Bahamas. For both COI and 16S, the results are similar if we pool populations undifferentiated by F_st_ in the Eastern and Southwestern regions (Table 4).

**Table 4:** Summary of regional genetic parameters for regions with multiple undifferentiated populations, including values of nucleotide diversity (π), Tajima's D_T_ and Fu's F_s_.

Gene	Region	N	*h*	π	D_T_	F_s_
**COI**	Southwestern	49	0.807	0.012	1.596	3.231
	Eastern	26	0.975	0.016	−1.401	−7.958**
**16S**	Southwestern	58	0.761	0.016	0.893	1.422
	Eastern	29	0.892	0.011	−0.0700	−2.141

**Note:** Significant p-values (p<0.05 and p<0.01) are indicated with one or two asterisks, and based on permutation test results.

Both Tajima's D and Fu's F_s_ were variable across sites, with both positive and negative values being observed (Table S1). In the Eastern Caribbean and Bahamas (BSS, WPR, USVI, BN), all values of both statistics were negative for both genes, with five of eight significantly so (Table S1). In contrast, values of both statistics were uniformly positive across both genes in three Southwestern sites (CR, PC, BO), although none of the tests were significant. For the Eastern region D_T_ was negative and near significance, while Fu's F_s_ was significantly negative (Table 4). For the Southwestern region, both test statistics were strongly positive albeit insignificantly so (Table 4).

## Discussion

In marine environments, genetic data can make an essential contribution to understanding population connectivity, as well as to conservation and management strategies, including the design of marine reserves. In this study, we present data on population connectivity from the commercially exploited *C. pica*, an important member of Caribbean rocky shore communities with limited dispersal potential. Several findings emerge from this data. First, a clear lack of differentiation was apparent in the small number of pairwise comparisons between sites separated by distances <300 km in both Eastern (WPR and USVI) and Southwestern (CR, PC and BO) regions. Connectivity within these regions was demonstrated by low and insignificant values of F_ST_ (Table 1) and higher frequencies of shared haplotypes (Figure 2). Recent studies of Caribbean currents suggest an average current speed of 1–2 km per hour and report that larval connectivity decreases past the 200 km barrier among Caribbean locations for coral reef fishes [34]. Even given the short duration of the lecithotrophic larval stage (4.5 days of development), *C. pica* larvae could travel up to 216 Km via inshore currents before settlement, and strong genetic connectivity on this spatial scale is not surprising. However, the limited number of small spatial scale comparisons also fall within the boundaries of connectivity regions defined on the basis of Caribbean hydrodynamics [9], and it is unknown whether comparisons of similar scale across hydrodynamic boundaries would indicate differentiation.

On larger scales, however, connectivity is generally low, as significant genetic differentiation is apparent across regions and between sites at both loci sampled. The patterns of differentiation in some cases do appear consistent with boundaries of connectivity regions in the Caribbean defined by hydrodynamic models. The majority of F_ST_ values among sites in different regions are significantly large. For example, the three Southwestern sites (CR, PC, BO) are strongly differentiated from the nearby Panama site (BV), in all analyses, consistent with the influence of coastal oceanography. The Panama-Colombia oceanographic gyre may be contributing to the isolation of Central Panama (BV) from other Central American sites. Previous studies suggested that regional current patterns from the northwestern coast of Panama to Belize might lead to mtDNA differentiation in coral reef fishes [15]. In the case of rocky intertidal species like *C. pica,* another important factor promoting differentiation between these regions is the absence of suitable rocky intertidal habitat in areas to the west of the Panama Canal on the Caribbean shore of Panama, which limits recruitment between central and western Panama.

However, the overall structure observed is not best explained by the four broad regions of isolation defined by hydrodynamic models of dispersal within the Caribbean [9], as an insignificant proportion of mitochondrial genetic variation at both loci sampled is partitioned among these regions (Table 3). This is shown by pairwise F_ST_ values that indicate differentiation within connectivity regions, and a lack of differentiation between some sites in different regions, and is supported by SAMOVA analysis which indicates alternative groupings of sites which maximize the among groups variance component. Two particular exceptions to the pattern expected from the hydrodynamic connectivity model are noted.

First, while the Bahamas is genetically distinct from most sites, as predicted by the model, it is not significantly differentiated at the COI locus from sites in the Eastern region, and is grouped with these sites in the SAMOVA analysis for 16S. The Antilles current (Figure 1) may mediate larval influx, primarily from the U.S. Virgin Islands to the Bahamas. Previous studies reported similarities of Bahamas sites such as San Salvador (BSS) to Eastern populations (WPR and USVI) mainly promoted by the northward flow of the Antilles current [53]. Genetic similarities between the same areas have also been observed for the elk horn coral (*Acropora palmata*) and in the goby *Elacatinus evelynae* [10], [11].

Secondly, while Bonaire (BN) falls within the eastern region defined by hydrodynamic models, the separation of Bonaire from the most of the other regions is supported by genetic data. This result contrasts with previous studies in corals in which populations from Bonaire or nearby Curacao group with other eastern populations in the context of an east-west genetic break in the Caribbean [11], [54]. For both mitochondrial loci, F_ST_ values are large and significant in nearly all pairwise comparisons involving this site, and SAMOVA supports Bonaire as a separate group for COI. Several private haplotypes were found for COI in Bonaire, supporting the isolation of this region in *C. pica*. Yet, values of F_st_ are reduced in comparisons with sites in the Southwestern region (CR, PC, BO) and in several comparisons are insignificant across both loci. For 16S, the structure that maximizes among-groups genetic variance places Bonaire with the Southwestern region sites. These results could be due to a more recent separation of these sites, or higher connectivity between them, possibly mediated by the Caribbean current.

A number of studies support the notion that the Caribbean region is a coherent marine biogeographic province with reduced or no phylogeographic barriers to promote genetic differentiation [55]. This is especially true for species with extended larval development such as the Caribbean queen conch [27], spiny lobster [30], *Diadema* and the red rock urchin, *Echinometra lucunter* [21], [56], [57] as well as coral reef fishes [15], [58]–[61]. Yet established genetic breaks in corals and fishes support the influence of common vicariant events on the biogeographic origin of Caribbean species [62]. These findings suggest that larval retention and self-recruitment induced by mesoscale oceanography, larval behavior, and oceanographic barriers promote elevated genetic structure [9], [10], [12], [54]. This study adds to this body of evidence, but also supports the notion that variation in patterns of population structure among taxa, such as the isolation of Bonaire in *C. pica*, is not always easily predictable by features of life-history such as PLD [8] or hydrodynamic models, and highlights the importance of additional genetic studies to support the construction of multispecies marine reserves.

Genetic diversity is an important indicator of the level of fitness and sustainability in marine populations, and *C. pica* populations exhibited moderate to high values of genetic diversity in the majority of analyzed sites. These values are similar to others reported for Caribbean, Atlantic and Pacific gastropods for mitochondrial genes [27], [63], [64]. Yet, sites in the eastern Caribbean tended towards higher values of genetic diversity. This pattern could be driven by large scale population expansion scenarios, or source-sink dynamics, but given the isolation of regions and the inconsistent results of neutrality tests, the pattern may simply be due to differences in the amount of suitable habitat in different areas of the Caribbean. Another possible explanation for the observed pattern is that sites such as Bonaire and the US Virgin Islands have conservation and management policies which might contribute to the high levels of genetic diversity [65]. The US Virgin Islands is the only territory within the eastern Caribbean region with a closed fishing season for topshell during its reproductive phase [32] and in the case of Bonaire, the whole island is managed as a marine reserve. In contrast, in Costa Rica and Panama *C. pica* is harvested for local consumption and sold as exotic handcraft to tourists, and a fishery ban for this species has not been established yet. Uniformly positive values of neutrality tests in this region may indicate a bottleneck in these populations, although it is unclear whether this result could be related to anthropogenic factors.

Access to genetic information and its integration into present management and conservation policies is only a reality for a small number of species. For *C. pica* the high degree of population structure, the existence of common connectivity areas among neighboring countries (<300 km), the partial concordance of these areas with previous areas established for coral and fishes and spatial trends in genetic diversity are important factors to consider in future conservation and management plans. Conservation partners can be established between areas of strong connectivity and reduced differentiation. In addition, the presence of areas of reduced diversity (Southwestern sites) can be used as scientific evidence for the future implementation of a fishery ban for *C. pica* and for replenishment protocols in these areas [3], [5].

## Supporting Information

Table S1 Summary of genetic parameters by population. Summary of genetic parameters include: geographic coordinates, GenBank accession numbers, number of COI and 16S sequences per location (N), number of observed haplotypes (H), haplotype diversity (h), nucleotide diversity (π), and neutrality tests: Tajima's DT and Fu's Fs. Note: Significant p-values (p<0.05 and p<0.01) are indicated with one or two asterisks based on permutation test results.
(0.01 MB DOCX)
