# Correction: Population Genetics of a Trochid Gastropod Broadens Picture of Caribbean Sea Connectivity

**DOI:** 10.1371/annotation/57fcda53-c9ef-486f-a87c-3cb3673cea70

**Published:** 2010-09-29

**Authors:** Edgardo Díaz-Ferguson, Robert A. Haney, John P. Wares, Brian R. Silliman

The middle initials of the second, third, and fourth authors were omitted. The correct names are Robert A. Haney, John P. Wares, and Brian R. Silliman. The citation should read: Díaz-Ferguson E, Haney RA, Wares JP, Silliman BR (2010) Population Genetics of a Trochid Gastropod Broadens Picture of Caribbean Sea Connectivity. PLoS ONE 5(9): e12675. doi:10.1371/journal.pone.0012675

