# Supplementary material for: Population Genetics of a Trochid Gastropod Broadens Picture of Caribbean Sea Connectivity
Source: PLoS One. 2010 Sep 10;5(9):e12675. doi: 10.1371/journal.pone.0012675 (PMC2937038; doi:10.1371/journal.pone.0012675)
Supplement: Table S1 — Summary of genetic parameters by population. Summary of genetic parameters include: geographic coordinates, GenBank accession numbers, number of COI and 16S sequences per location (N), number of observed haplotypes (H), haplotype diversity (h), nucleotide diversity (π), and neutrality tests: Tajima's DT and Fu's Fs. Note: Significant p-values (p<0.05 and p<0.01) are indicated with one or two asterisks based on permutation test results. (0.01 MB DOCX) [file pone.0012675.s001.docx]

| **Gene** | **Location Name and Code** | **Geographic Coordinates** | **GenBank Acc.Num.** | **N** | ***h*** | **π** | D_T_ | F_s_ |
| --- | --- | --- | --- | --- | --- | --- | --- | --- |
| **COI** | 1.Bahamas (**BSS)** | **24.06N, 74.29W** | GU726381-  GU726389 | 9 | 0.972 | 0.015 | -0.458 | -2.379 |
|  | 2.Punta Cocles Costa Rica (**CR)** | **10.45N,83.20W** | GU726390-GU726412 | 23 | 0.810 | 0.0127 | 1.725 | 4.348 |
|  | 3.Playa Chiquita, Costa Rica (**PC)** | **9.38N, 82.42W** | GU726413-GU726419 | 7 | 0.905 | 0.014 | 1.106 | 0.419 |
|  | 4.Bocas del Toro, Panama **(BO)** | **9.17N, 82.08W** | GU726420-GU726438 | 19 | 0.784 | 0.013 | 0.807 | 1.406 |
|  | 5.Buenaventura, Panama (**BV)** | **9.31N,79.40W** | GU726439-  GU726450 | 12 | 0.924 | 0.010 | 0.166 | -0.737 |
|  | 6. West Puerto Rico (**WPR)** | **18.25N, 67.09W** | GU726470-GU726486 | 17 | 0.971 | 0.017 | -0.656 | -3.854* |
|  | 7.US Virgin Islands (**USVI)** | **17.41N,64.41W** | GU726461-GU726469 | 9 | 0.972 | 0.016 | -1.1442 | -2.23 |
|  | 8. Bonaire (**BN)** | **12.12N, 68.16W** | GU726451-  GU726460 | 10 | 0.933 | 0.010 | -1.186 | -1.510 |
| **16S rRNA** | 1.Bahamas **(BSS)** | **24.06N, 74.29W** | GU733509-GU733523 | 15 | 0.895 | 0.006 | -1.307 | -6.101** |
|  | 2.Punta Cocles Costa Rica **(CR)** | **10.45N,83.20W** | GU733544-GU733560 | 17 | 0.581 | 0.012 | 0.457 | 2.800 |
|  | 3.Playa Chiquita, Costa Rica **(PC)** | **9.38N, 82.42˚W** | GU733561-GU733579 | 19 | 0.83 | 0.017 | 1.539 | 2.187 |
|  | 4.Bocas del Toro, Panama  **(BO)** | **9.17N, 82.0W** | GU733494-GU733508  GU733524-GU733530 | 22 | 0.801 | 0.017 | 1.272 | 2.639 |
|  | 5.Buenaventura, Panama **(BV)** | **9.31N,79.40W** | GU733531-GU733543 | 13 | 0.846 | 0.004 | -0.754 | -3.156** |
|  | 6.West Puerto Rico (**WPR)** | **18.25N, 67.09W** | GU733580-GU733593 | 14 | 0.934 | 0.014 | -0.106 | -1.469 |
|  | 7.US Virgin Islands (**USVI)** | **17.41N,64.41W** | GU733482-GU733493  GU733605-GU733607 | 15 | 0.819 | 0.008 | -1.638* | -0.779 |
|  | 8. Bonaire **(BN)** | **12.12N, 68.16W** | GU733605-GU733607 | 11 | 0.982 | 0.017 | -1.548* | -3.901* |
